# A Descriptive Analysis of Mass Shootings in the United States From 2010 to 2020: The Relationship Between Firearm Dealership Density and Proximity to Mass Shooting Sites and a Comparison With McDonald's and Starbucks Retailers

**DOI:** 10.7759/cureus.29302

**Published:** 2022-09-18

**Authors:** Iordanis Zakopoulos, Karan Varshney, Jon T Macy, Russell K McIntire

**Affiliations:** 1 College of Population Health, Thomas Jefferson University, Philadelphia, USA; 2 Surgery, Memorial Hermann/University of Texas Health Science Center at Houston, Houston, USA; 3 School of Public Health, Indiana University Bloomington, Bloomington, USA

**Keywords:** united states mass shooting, public health, violence, population density, firearm dealerships, mass shootings

## Abstract

The public health community needs to better understand the complex factors that contribute to mass shootings in the United States (US). We explored how firearm dealership density related to geographic distance from mass shooting sites in the US in 2010-2020, and compared it with the corresponding density of Starbucks (Starbucks Corporation, Seattle, Washington, United States) and McDonald’s (McDonald's Corporation, Chicago, Illinois, United States) outlets. We obtained locations of firearm dealerships, Starbucks, and McDonald's retailers, as well as mass shootings across the contiguous US from 2010 to 2020. We mapped buffer rings, at 1, 5, 10, 30, and 50 miles around the locations of each mass shooting. We compared the per area and per population density of the dealerships and the two types of retailers around mass shooting sites within each buffer ring. We identified 67 mass shootings from 2010-2020. We mapped 54,722 firearm dealerships, with 28.1% located within 50 miles of mass shootings. Within a 50-mile radius of shooting events, there were an average of 0.30 dealerships, while there were only 0.14 Starbucks and 0.12 McDonald’s outlets per 10 square miles. There was an incremental reduction in the density of firearm dealerships, Starbucks, and McDonald’s as the distance from the mass shooting site increased. Density per population did not show a similar relationship. Our findings suggest that the availability of firearms deserves more study as a contributing factor to mass shootings in the US. The high number, area, and population density of firearm dealerships compared to the other two retailers illuminate how numerous firearm dealerships are compared to common retailers in the US.

## Introduction

Gun violence resulted in 39,707 deaths in the United States (US) in 2019 [1], which marked a 17% increase since 1999 [2]. Although public mass shootings accounted for less than 0.1% of homicides in the US between 2000 and 2016, the rate at which mass shootings occur has tripled from 2011 to 2014, and more recently the number of mass shootings increased from 417 in 2019 to 611 in 2020 despite the COVID-19 pandemic [3,4]. Another analysis conducted by Everytown for Gun Safety Support Fund found that between 2009 and 2020, 1,363 people were killed in 240 mass shootings in the US [5]. However, mass shooting-related mortality is a poorly researched and understood subject. One reason for this is that a widely accepted definition for a public mass shooting has not been established, and different organizations and law enforcement agencies have provided varying definitions over time. The US Congressional Research Service defines the term “public mass shooting” as an event in one or more public places involving the murder with firearms of four or more people, excluding the shooter(s), selected indiscriminately within one event without a means to an end (e.g., terrorism, robbery) [6]. This also reflects the Federal Bureau of Investigation's 2005 definition of the term “mass murder” as the murder of four or more people [7]. In January 2013, a mandate by the US government lowered that baseline to three or more fatalities [8]. Follman et al. combined the above definitions and constructed an open-source database intending to study public mass shootings specifically, rather than gun violence as a whole, as a social phenomenon [9]. They focused on indiscriminate rampages in public places, excluding shootings stemming from more conventionally motivated crimes such as armed robbery or gang violence in which the perpetrators have not been identified. Based on their definition, as of February 2020, there had been 118 mass shootings in the US in the past four decades, most of which involved legally obtained firearms [10].

Easy access to firearms, whether legal or illegal, is one of the main drivers of gun violence. This relationship has been previously established by several studies [11-15]. For example, after analyzing the Centers for Disease Control and Prevention's Web-Based Injury Statistics Query and Reporting Systems (WISQARS™) database with data from all 50 states spanning three decades (1981-2010), Siegel et al. found that gun ownership was a significant predictor of firearm homicide rates at the state level [11]. Their model indicated that for each percentage point increase in gun ownership per state, the corresponding firearm homicide rate increased by 0.9%. In addition, several prior studies used cross-sectional analyses to detect a positive relationship between firearm homicide and/or suicide and gun ownership rates at different levels, such as neighborhood [12], county [13], and state [14]. Studies have shown that this relationship applies not just to homicide rates, but also to mass shootings, as investigated at the ecological level. Reaping et al. showed that US states with weaker gun laws and higher gun ownership rates had higher rates of mass shootings [15].

Legally operating dealerships are one source of firearms supply and the focus of the current study. According to the Bureau of Alcohol, Tobacco, Firearms, and Explosives (ATF), as of January 10, 2020, there were over 53,000 firearm dealerships across the US (federal firearms license (FFL) type 1, not including other FFL types or online sellers) [16]. Chao et al. found a correlation between statewide total FFL density and firearm-related mortality, which was stronger for deaths caused by suicide [17]. In addition, there have been certain press reports about firearm dealerships being more abundant than common commercial retailers such as groceries, Starbucks (Starbucks Corporation, Seattle, Washington, United States), and McDonald’s (McDonald's Corporation, Chicago, Illinois, United States) [18].

However, there is a paucity of research that investigates links between firearm dealership density and mass shootings. In the current study, we explored how firearm dealership density varied by geographic distance from the sites of mass shootings in the US and also compared it with the densities of two other major retail establishments, Starbucks and McDonald’s. We chose Starbucks and McDonald's retailers due to how widely abundant they are in many geographical settings. First, we compared the FFL type 1 firearm dealership density (by area and by population) within 50 miles of US mass shooting events between January 2010 and February 2020 to density measures for the whole US. Second, for distances within 50 miles, we explored how area dealership density and dealerships per population varied with proximity to the shooting sites. Finally, we analyzed similar data for two other types of common American commercial stores, Starbucks and McDonald’s, to compare their densities in relation to distance from mass shooting sites to that of firearm dealerships. These analyses with the Starbucks and McDonald's stores provide a point of comparison with firearm dealerships, and offer additional insights regarding the overall spatial distribution of mass shooting events.

## Materials and methods

Study sample

For this study, we obtained the locations of 67 major mass shooting events that occurred between August 2010 and February 2020 from the Mother Jones open-source online database [10]. The database included indiscriminate rampages in public places involving four or more deaths, excluding the attacker, for events up to and including December 31, 2012, and three or more deaths, excluding the attacker, for events beginning January 1, 2013 [9]. Shootings stemming from more conventionally motivated crimes, such as armed robbery or gang violence, or involving unidentified perpetrators were excluded from the database. 

Measures

**Figure 1:**
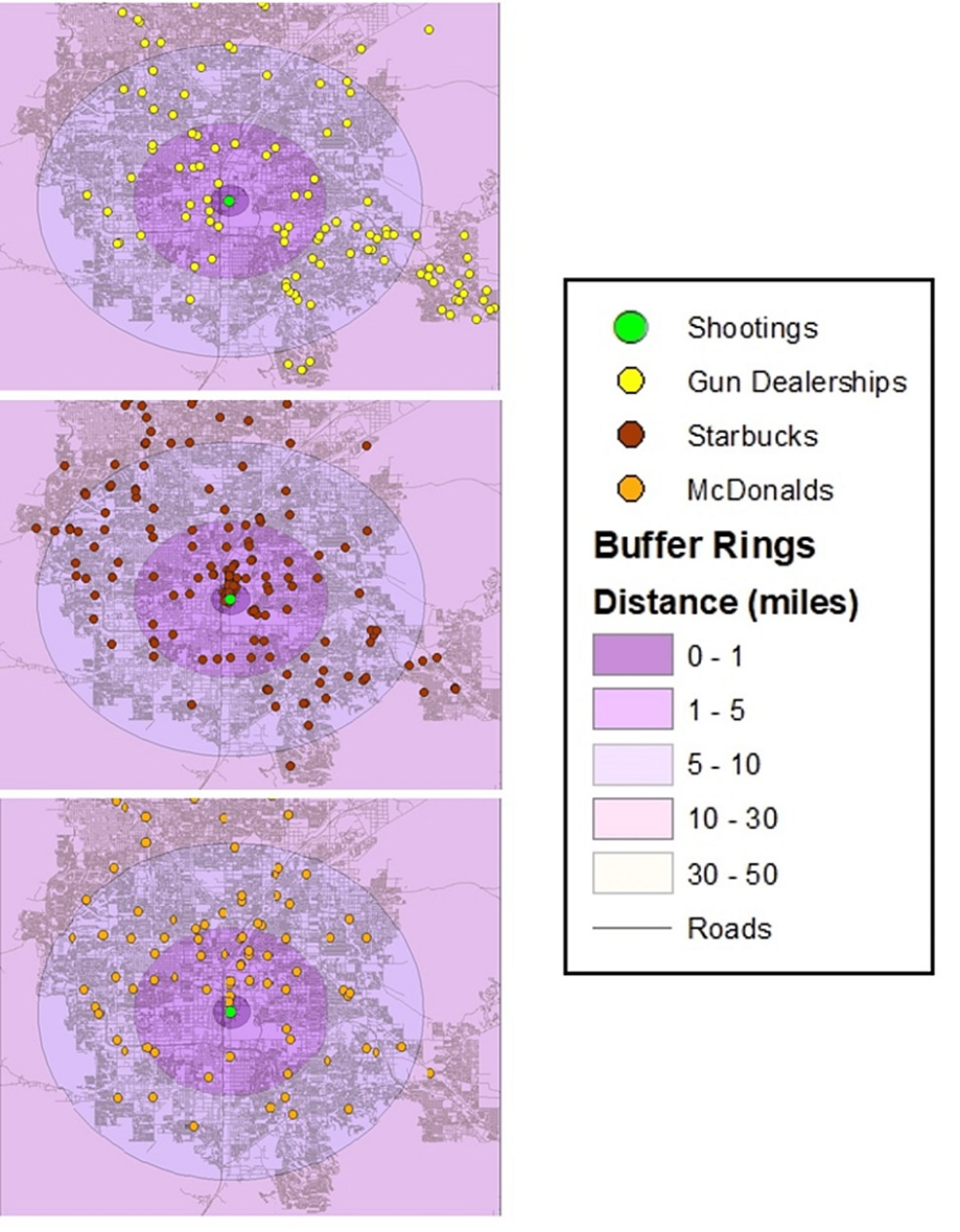
Maps of Las Vegas, Nevada, with buffer rings of 1, 5, 10, and 30 miles (50 not visible in image) around the mass shooting on October 1, 2017, each depicting point data of firearms dealerships, McDonald's outlets, and Starbucks stores Data source: Bureau of Alcohol, Tobacco, Firearms and Explosives (2015), ArcGIS (2014, 2016), Nevada Department of Transportation [16,20-22].

We used ArcGIS Desktop version 10.3.1 (Released 2015; Esri, Redlands, California, United States) to represent locations of mass shootings as points using the longitude and latitude coordinates provided by the database. We sourced firearm dealership addresses from ATF’s federal firearms listings 2015 data [16]. We chose 2015 as the best dataset to identify firearm dealerships because it represented the midpoint of the 10-year time span of the mass shootings of interest. We only included FFL type 1 gun dealerships, which are defined as “dealers in firearms or other than destructive devices (includes gunsmiths)" [19]. We did not consider the following for this study: pawnbrokers of firearms, dealers/pawnbrokers of destructive devices, or manufacturers/importers of ammunition/firearms/destructive devices. In addition, we mapped all Starbucks and McDonald’s retailers across the contiguous US. Both the Starbucks and McDonald’s data were sourced from ArcGIS Online's feature layers, and last updated in May 2014 (ArcGIS.com, 2014) and 2016 (ArcGIS.com, 2016), respectively [20,21]. While we recognize that the store total may have changed since this time period, we were unable to adjust for this in our analyses. All facilities were mapped as point features. We cleaned the addresses and geocoded them using ArcGIS Online’s World Geocoding Service (match rate over 99%). In total, we mapped 54,722 firearm dealerships, 11,609 Starbucks establishments, and 13,813 McDonald's locations in the US mainland. We examined how the density of dealerships and dealerships per population changed within the 50 miles radius around the shooting locations. Using ArcGIS, we drew five classes of buffer rings: 1, 5, 10, 30, and 50 miles around the locations of each mass shooting. For example, Figure 1 depicts the Las Vegas shooting in October, 2017 [22]. 

Analyses

Density by Area

We calculated the cumulative area of each buffer ring, in square miles, around each shooting by using the circle area formula π x R2, where R represents the buffer ring radius, and multiplying by 67, the total number of major mass shootings between 2010 and 2020 [9]. To calculate the mean dealership density for each buffer ring class, we divided the total count of facilities located in all 67 buffer rings of the same class by the cumulative area (in square miles) of the corresponding ring. 

Density per Population

We calculated the total population contained in each buffer ring using the US Census Bureau data (2010) [23]. We summed the total population of the census tracts with centroids contained in each buffer ring. These population numbers were used to determine the mean population density within each buffer ring (total population within each buffer divided by area of each buffer), and the total number of facilities (by facility type) per 100,000 population within each buffer ring.

## Results

There were 67 mass shootings in the contiguous US during 2010-2020. We identified 54,722 firearm dealerships across the contiguous US and the District of Columbia in 2015. This number, divided by the total area of the contiguous US (2,959,064.44 square miles), corresponded to 0.18 dealerships per 10 square miles, the national mean firearms dealership density.

Of the 54,722 firearm dealerships, 28.1% were within 50 miles of the 67 major mass shooting sites. Within this 50-mile radius surrounding the mass shootings, which encompasses an area of 525,950 square miles total, the density of firearm dealerships was 0.03 dealerships per square mile, 62.7% higher than the national mean firearms dealership density.

Regarding density per population, the national average for firearm dealerships was 17.69 dealerships per 100,000 population. Within 50 miles of major mass shootings, there were 10.63 dealerships per 100,000 population, which was 39.91% less than the national average.

Table 1 depicts the facilities per area, and facilities per population for firearm dealerships, Starbucks, and McDonald’s within each buffer ring.

**Table 1:** Per area and per population density of firearm dealerships, Starbucks stores, and McDonald’s outlets by distance from mass shooting sites in the United States, 2010-2020. Data source: Bureau of Alcohol, Tobacco, Firearms and Explosives (2015), ArcGIS (2014, 2016) [16,20-21].

Buffer Ring (miles)	Firearm Dealerships				Starbucks			McDonald’s		
	Count (%)	Per 10 sq miles		Per 100,000 population	Count (%)	Per 10 sq miles	Per 100,000 population	Count (%)	Per 10 sq miles	Per 100,000 population
0-1	75 (0.1)	3.60	9.75		149 (1.3)	7.15	19.37	64 (0.5)	3.07	8.32
1-5	798 (1.5)	1.60	6.49		891 (7.3)	1.79	7.24	496 (3.6)	0.99	4.03
5-10	1653 (3.02)	1.00	6.40		1,347 (11.6)	0.81	5.22	1,037 (7.5)	0.63	4.02
10-30	6979 (12.8)	0.40	9.62		3,394 (29.2)	0.19	4.68	2,839 (20.6)	0.16	3.91
30-50	5896 (10.8)	0.20	17.61		1,162 (10.0)	0.04	3.47	1,527 (11.1)	0.05	4.56
0-50	15,401 (28.1)	0.30	10.63		6,943 (59.8)	0.14	4.79	5,963 (43.2)	0.12	4.12

**Figure 2:**
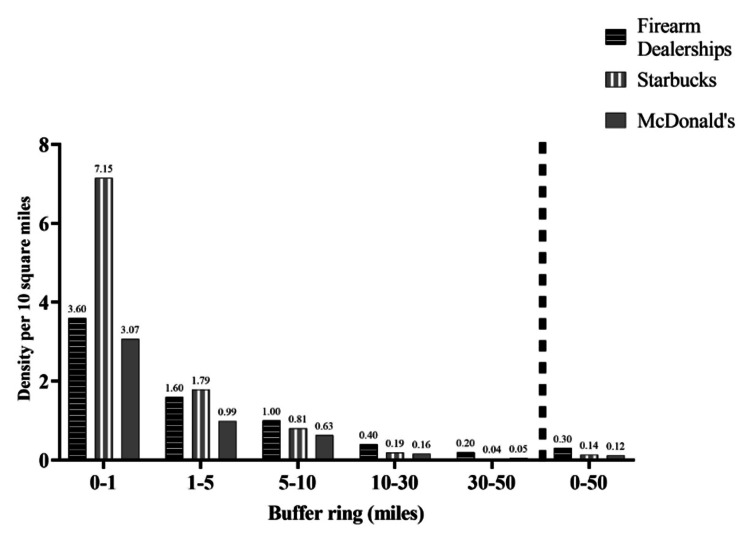
Mean density of firearm dealership, Starbucks stores, and McDonald’s outlets (per 10 sq miles) per buffer ring around the sites of major United States mass shootings, 2010-2020. Data source: Bureau of Alcohol, Tobacco, Firearms and Explosives (2015), ArcGIS (2014, 2016) [16,20-21].

As shown in Table 1 and Figure 2, we observed an incremental reduction in dealership density as the distance from the location of the mass shooting increased, from the closest buffer ring to the furthest buffer ring. Within 1 mile of all 67 mass shooting sites, the dealership density was 3.60 dealerships per 10 square miles, which is 19 times higher than the national average. In the next three successive rings, the density ratios were 1.6, 1.0, and 0.4 dealerships per 10 square miles, which is eight, six, and two times the national average, respectively. In the last buffer ring (30-50 miles), the density began to resemble the national average, with the dealership density at 0.2 per 10 square miles. 

Due to the urban nature of the vast majority of mass shooting sites, population density decreased sharply as distance from the mass shooting event increased. The highest population density per square mile was within 1 mile from mass shootings, at 3653.59 people per square mile. Between 30-50 miles from shootings, the mean population density was lowest at 99.40 people per square mile.

Starbucks and McDonald’s restaurant density exhibited similar patterns as firearm dealership density, decreasing as the distance from sites of mass shootings increased. However, both the number of firearm dealerships (15,401), and the density (0.30 dealerships per 10 square miles) within 50 miles from the sites of mass shootings was higher in comparison to Starbucks (6,943 retailers; 0.14 per 10 square miles) and McDonald’s (5,963 retailers; 0.12 per 10 square miles) respectively.

**Figure 3:**
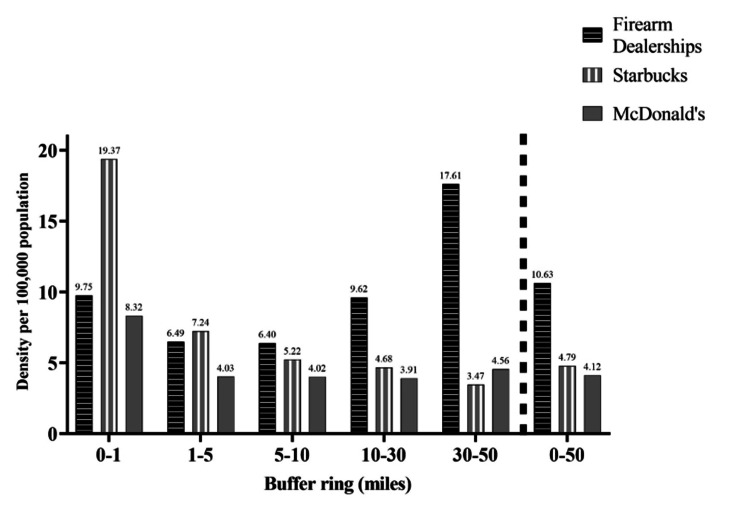
Mean density of firearm dealerships, Starbucks stires, and McDonald’s outlets (per 100,000 population) per buffer ring around the sites of major United States mass shootings, 2010-2020. Data source: Bureau of Alcohol, Tobacco, Firearms and Explosives (2015), ArcGIS (2014, 2016) [16,20-21].

Both the number of Starbucks and McDonald’s retailers per 100,000 population decreased as the distance from the site of mass shootings increased. Starbucks was 19.37 per 100,000 population within 1 mile, and 3.47 per 100,000 population between 30-50 miles from the site of mass shootings. McDonald’s density was 8.32 per 100,000 population within 1 mile, and 4.56 per 100,000 population between 30-50 miles from the site of mass shootings. While Starbucks and McDonald’s stores per 100,000 decreased sharply as the distance from the site of mass shootings increased, this was not the case for firearm dealerships; firearm dealerships per 100,000 population did not decrease incrementally as distance from the site of mass shootings increased. As shown in Figure 3, dealerships per population was highest in the 30-50 mile buffer ring (17.61 per 100,000), followed by the 0-1 mile ring (9.75), 10-30 mile ring (9.62), 1-5 mile ring (6.49), and 5-10 mile ring (6.40).

## Discussion

This study examined the relationship between firearm dealer density and the location of mass shootings in the contiguous US between 2010 and 2020. For comparison, the densities of two other widely recognized retailers, Starbucks and McDonald’s, were similarly analyzed. Within 50 miles of the mass shooting sites, the number and density of firearm dealerships per area and per population were more than double those of either Starbucks or McDonald’s. Specifically, there were 15,401 firearm dealerships compared to 6,943 Starbucks and 5,963 McDonald’s. Another important finding was that areas closer to mass shootings had higher firearm dealership density per square mile. As the distance from mass shootings increased, both the absolute number of dealerships and the dealership density within each buffer decreased. The same area density relationships were found for Starbucks and McDonald’s. 

Although area-based firearm dealership density was clearly related to distance from the sites of mass shootings, dealerships per population showed differing trends. Population density decreased as the distance from the location of mass shootings increased. This makes sense, given that the vast majority of mass shootings took place in urban areas, with higher population density compared to suburban and rural areas. In comparison, the number of Starbucks and McDonald’s stores per 100,000 population notably decreased as the distance from mass shooting sites increased. However, the pattern for firearm dealerships per 100,000 population showed an increase, starting from 10 miles away from the mass shooting site. The one striking difference worth noting is that in the buffer ring 30 to 50 miles from the mass shooting sites, there were 17.61 firearm dealerships per 100,000 population compared to 3.47 for Starbucks and 4.56 for McDonald’s. 

These findings suggest that access to firearms may correlate with the occurrence of mass shootings. Our area density results are consistent with other studies that have shown that access to firearms correlates with firearm injuries at the population level [12-15]. Our findings, in line with other ecological analyses, did not attempt to identify an association between each mass shooting site and its corresponding surrounding dealership density. Rather, we grouped density data for all sites for each class of buffer rings and examined the mean density of each ring and the distance of each ring from the mass shooting site. Similar to previous studies [24,25], our analysis strategy followed the assumption that the number and density of FFL in a region are reflective at some degree of the prevalence of firearms. On the same note, Stansfield and Semenza argued that the presence of dealerships results in a higher likelihood of an available firearm being acquired and used by a potential intimate partner violence perpetrator, immediately after or during an altercation [24]. Another pathway to explain how firearm dealerships might affect crime was proposed by Steidley et al., who argued that the presence of FFL stores fuel local crime (homicide and robbery) both directly by providing opportunities for crime and indirectly by transforming neighborhood perceptions and weakening social control efforts [26].

Our study is not without limitations. First, the areas used to calculate density did not account for different zoning classifications (i.e. commercial, residential, industrial) or inaccessible areas (e.g. lakes, mountains, areas outside the US border). Such considerations might alter the results depending on the zoning and unique characteristics of the location surrounding each mass shooting. However, all results are estimates calculated based on the same assumptions as described above and are comparable to one another. Second, only FFL type 1 dealerships from 2015 were analyzed, as this year was the midpoint of shooting events occurring between 2010-2020; similarly, we relied on data from 2016 and 2014 for McDonald's and Starbucks retailers, respectively, which is a limitation. Given the cross-sectional nature of the analysis, we cannot identify a temporal relationship between access to dealerships and mass shooting events. The relationship identified may indicate that easier access to gun dealerships precedes mass shooting events or that a given mass shooting event might cause a higher demand for firearms locally for personal safety, and thus increase the number (and density) of retailers. The limitations of the data and study design preclude us from making any statement of a causal relationship between dealership density and mass shootings. 

Lastly, the association between dealership density and mass shootings may be confounded by several other variables, such as population distribution, educational status, and income level. Wiebe et al. provided evidence of an association between the per capita rate of licensed firearm dealers in a county and the rate of firearm homicide [25]. A higher number of FFLs was associated with significantly higher rates of firearm homicide in major cities. No relationship was found for small towns, while an inverse association was described for small cities and suburbs [25]. Moreover, Steidley et al. indicated that after weighting by census tract population, homicide and robbery rates increased by the number of firearm dealerships in a neighborhood [26]. This relationship persisted even after controlling for crime-related factors at both the census tract and metropolitan level and regardless of dealership type (except for big box stores).

Mass shootings are only one instance of the gun violence epidemic, which also includes gun-related crime, domestic violence, accidents, and firearm-assisted suicide. Our research can add to the conversation on firearm policy at the local, state, and federal levels, and provide a rationale for local ordinances aimed at reducing the density of firearm dealerships, which is not without precedence [27]. Legal and community-based policies, such as shutting down inactive or corrupt gun dealerships, and enacting domestic violence firearm restrictions, have previously been proposed as solutions to widespread access to firearms [25,28]. Increased enactment of such policies in localized jurisdictions may be effective in reducing firearm access, particularly to high-risk individuals.

While we did not quantify a statistical association between firearm dealership density and mass shootings, we did identify a descriptive inverse relationship between distance from the shooting sites and dealership density. We also demonstrated a substantially higher density of firearm dealerships both in terms of person square mile and in terms of per population terms compared to Starbucks and McDonald’s locations within 50 miles of mass shooting sites. This is important for a number of reasons. First of all, this comparison with Starbucks and McDonald’s retailers relative to mass shootings highlights the pervasiveness of firearm dealerships in areas where mass shootings have occurred. Secondly, this comparison more generally demonstrates how widespread firearm dealerships are across the US. Furthermore, these analyses provide guidance for future research to determine what temporal relationships do, and do not, exist in relation to mass shooting sites.

Future research should quantify these relationships and identify if any temporal relationship exists between firearm dealerships and mass shootings. Further, the sheer number and density of firearm dealers that we identified in the US could support arguments advocating for zoning restrictions on firearm dealership density and, possibly, dealerships’ proximity to schools, liquor-selling establishments, and residential areas. There are instances where zoning laws have been shown to reduce rates of neighborhood crime [29], indicating that gun-free zoning laws developed based on neighborhood consensus can lead to violence reduction [30]. Our findings add to a body of evidence that could help to justify land-use legislation for the interest of public health and safety. 

## Conclusions

Our findings show both that firearm dealership total, and firearm dealership density, within 50 miles of mass shooting sites are higher compared to McDonald's and Starbucks retailers. Furthermore, it was demonstrated that the density of dealerships was higher closer to locations of mass shootings. While the cross sectional and ecological nature of our study design precludes us from making statements about causation, our results provide descriptive rationale to further explore relationships between firearm dealerships and mass shootings. More evidence is needed to decipher the causes of mass shootings in order to generate aimful and evidence-based policy action to reduce the frequency of these violent acts.
